# Between “normality” and diagnosis: strains between the *I* and the *Me* in undiagnosed adolescents with ADHD symptoms

**DOI:** 10.3389/fpsyt.2026.1771510

**Published:** 2026-05-25

**Authors:** Jan Grimell, Lies Youcefi, Matilda A. Frick

**Affiliations:** 1 Department of Sociology, Faculty of Social Sciences, Umeå University, Umeå, Sweden; 2 Uppsala Universitet Sociologiska Institutionen, Uppsala, Sweden; 3 Stockholm Universitet Psykologiska Institutionen, Stockholm, Sweden; 4 Department of Medical Sciences, Uppsala University, Uppsala, Sweden

**Keywords:** ADHD, dialogue, *I*, medicalization, self, undiagnosed, youth

## Abstract

Adolescents who display ADHD-like difficulties but remain undiagnosed occupy a diagnostic borderland in which medical, social, and institutional expectations collide. In the Swedish context, where rates of diagnosed ADHD have risen sharply, this constitutes an understudied and increasingly marginalized group. Drawing on Mead’s concepts of the *I* and the *Me*, the study explores how ten Swedish adolescents negotiated their emerging selves while positioned between “normality” and diagnosis. Based on in-depth qualitative interviews and an abductive analytical strategy, the analysis shows that parents, teachers, peers, and digital media frequently activated medicalized interpretations of the adolescents’ behaviors. Participants moved between spontaneous expressions of the *I* and socially regulated *Mes*, often experiencing tension when their actions diverged from institutional, familial, or peer expectations. A recurring pattern was a form of double stigma: first, adolescents were judged for behavioral deviations that were not recognized as legitimate symptoms; second, when they received extra support or accommodations in school, these measures were sometimes viewed by peers as unfair due to the absence of a formal diagnosis. Some participants resisted stereotyped images of ADHD, whereas others saw diagnosis as a potential source of recognition, explanation, or legitimacy. Overall, the findings illustrate how medical discourse structures the very terrain on which the self emerges for adolescents positioned outside formal diagnostic boundaries. They also speak to a central concern within clinical psychiatry and diagnostic research on ADHD: as diagnostic frameworks increasingly govern how deviance, normality, and support needs are defined, those who do not neatly align with established categories risk having their experiences misread, marginalized, or left without adequate recognition and support. Further research is needed to examine the prevalence of these dynamics as well as to deepen qualitative understanding of adolescents who inhabit this diagnostic gray zone.

## Introduction

During adolescence, the question “Who am I?” becomes particularly salient. It is a period in which individuals develop a more differentiated understanding of themselves and begin the task of locating the self within a broader cultural and social landscape (1–3). Any answer to the question “Who am I?” is grounded in the individual’s interpretation of themselves in relation to the social world (4, 5). This requires both introspection and outward engagement—that is, drawing on personal experience while simultaneously interpreting feedback from the social environment. Through this *looking-glass self* process, individuals integrate information about themselves conveyed by others (4), and in doing so, the self emerges.

The emerging self can be understood as an ongoing interplay between what 5 conceptualized as the *I* (the spontaneous, creative, and unique aspects of the person) and the *Me* (the internalized values, norms, and roles of society). According to Mead, all individuals are born with a spontaneous and distinct *I*, which represents originality, creativity, and the primordial aspects of human agency. At the same time, the individual is, from birth, embedded within a cultural and social context whose moral codes, expectations, and role structures begin to socialize the spontaneous *I*. Through this process, the *Me* disciplines the *I* in accordance with socially reproduced norms and expectations (5).

Adolescents who find it difficult to be guided or regulated by the *Me*, and who struggle to transition from rudimentary role play to the more sophisticated mastery of role games (5), may encounter substantial challenges in the emerging self. These difficulties may also lead to potentially negative experiences. For young people who repeatedly experience stigma—being negatively treated because of perceived functional, behavioral, or social deviance—the question of who they are may become especially fraught (6). These individuals risk becoming caught between the *I* and the *Me* in ways that obstruct the emerging self, and may even deeply complicate its dialogical capacities (7).

This tension may be particularly taxing among adolescents exhibiting behaviors commonly linked to what is clinically defined as attention-deficit/hyperactivity disorder (ADHD). That is, enduring and debilitating difficulties with attention, concentration, impulsivity, and/or hyperactivity. Positioned between social expectations of “normality” and the categorization of deviancy and pathology implied by diagnosis, these adolescents often experience a strained interplay between *I* and *Me*. A clinical diagnosis may alter societal perceptions of deviant behavior, performance, and role-taking: through diagnosis, behaviors previously defined as deviant become recast as “normal,” typical, or at least expected, within the diagnostic frame (8–11). At the same time, society offers medically and pedagogically oriented interventions intended—at least theoretically—to ease the conflict between *I* and *Me*.

Research on adolescents situated in this liminal space between normality and diagnosis remains limited. Existing scholarship has largely focused on the lived experience of youth with a clinical ADHD diagnosis (12–20). This gap is amplified in the Swedish context, where ADHD diagnoses have increased dramatically over the past decade (21, 22). Consequently, adolescents who display ADHD behaviors but for various reasons remain without a formal diagnosis constitute an overlooked group, leaving knowledge and understanding of their experiences markedly limited.

The aim of this article is therefore to explore how the emerging self—conceptualized through 5—was shaped by the experiences of ten Swedish adolescents positioned between normality and diagnosis. All participants reported clear ADHD-related symptoms but had not, at the time of the interviews, received a formal clinical ADHD diagnosis.

### The medical and social dynamics of ADHD

Clinical descriptions of ADHD phenomena have existed for more than 200 years (23) and are currently most prominently noted in the fifth edition of the *Diagnostic and Statistical Manual of Mental Disorders* (DSM-5) (24). Clinically, ADHD is characterized by pervasive, debilitating and developmentally inappropriate levels of inattention, impulsivity, and hyperactivity. The global prevalence of ADHD is estimated to approximately 5-7% among children and adolescents (25), although current figures in Sweden and elsewhere exceed these estimates (26). There is no biological marker or specific test for ADHD. According to international guidelines, a diagnosis of ADHD is made by an experienced clinician based on a thorough medical and developmental history, and observer reports (usually by parents and teachers) including rating scales and a diagnostic interview (27). Throughout history, ADHD phenomena have been conceptualized within a variety of competing ontological discourses, each shaping its moral status in different ways (28). These ontological discourses form part of the broader construction of how ADHD phenomena are understood, and although contemporary discourse is dominated by medical framings, there is ongoing disagreement about which narratives should underpin our understanding of ADHD (29).

Overall, research across disciplines has highlighted the hegemonic position of medicalized understandings within contemporary health and welfare systems. Critique has emerged not along a simple medical versus social science divide, but from a range of critical and non-medicalized perspectives—including within medicine itself—questioning the normative, institutional, and epistemological assumptions underpinning medicalization (8, 9).

One central locus of such critique concerns psychiatric diagnoses, where medicalization is enacted primarily through symptom-based classification rather than through clearly defined organic pathology. As is characteristic of contemporary psychiatric diagnosis more broadly, psychiatric diagnoses—including ADHD—are defined through symptom-based classification rather than by reference to any specific, necessary, and sufficient organic pathology. According to Brante (30), this reliance on symptom-based criteria contributes to ongoing debates about the scientific foundations of psychiatric diagnoses in general and ADHD in particular. Lassinantti (10) considers ADHD a contested diagnosis, given the uncertainty about whether it should be understood as a psychological, social, or biological phenomenon. Ophir (31) highlights the dominance of the medical framework but also questions its scientific validity, arguing that ADHD is not necessarily an objective, chronic neurological condition, but rather a modern phenomenon in which behaviors are medicalized, particularly in relation to school-related performance demands. Unsurprisingly, social science research foregrounds social and sociocultural perspectives on the origins, experience, and management of ADHD. Horton-Salway & Davies (28), for example, argue that ADHD is a social and discursive product and instead emphasize a social constructionist approach to understanding and addressing ADHD. Not to leave out, contemporary perspectives of neurodiversity acknowledge that neurodevelopmental conditions such as ADHD are not categorical in nature and do not clearly differentiate between diagnosed or undiagnosed individuals (32). The neurodiversity movement often embraces the diagnostic labels, yet reject the notion that they are disorders caused by brain dysfunction. Instead, they stress that the conditions arise due to a mismatch between the individual and their environment (32). The neurodiversity perspective is embraced by parts of the medical establishment, and as such the dichotomy between the medical discourse and social perspectives are not always clear-cut.

The present study takes as its starting point the notion that ADHD is a phenomenon positioned within a medical discourse through which it gains legitimacy. Simultaneously, the ADHD phenomenon and its legitimizing medical discourse interacts with one’s experience of their own functioning and self, as well as with how others perceive the individual. “Normality” would then be conceptually understood as the antithesis to what is commonly referred to as ADHD. Normality needs to be conceptually understood through its narrative role in defining oneself, its dynamic relations to concepts of deviancy, and its relation to the normate (33). Within the scope of this article, adolescents who do not experience nor exhibit behaviors linked to enduring and debilitating difficulties with attention, concentration, impulsivity, and/or hyperactivity constitute the normate.

A medical ontology of ADHD implies specific pathological deviations in the physical body that manifest as symptoms such as hyperactivity and difficulties concentrating, and which can therefore be alleviated through particular forms of medication (9, 29). The diagnostic manual DSM—sometimes described as the authoritative voice of psychiatry (30)—initially had a more psychoanalytic orientation, but with the rise of psychiatry it has become increasingly categorical and medically oriented. Through psychiatric authority, the DSM has become a tool for reducing diagnostic uncertainty and creating clear categories for streamlined assessment, diagnosis, and treatment. This process has also contributed to the invisibilization of certain groups, since DSM classifications are derived primarily from studies conducted on majority populations. Within a social explanatory framework, however, the origins, experiences, and appropriate responses to ADHD-related difficulties look entirely different (34). This critique is rooted in a broader discussion of psychiatric diagnoses being assigned for behaviors that may be perfectly natural variations in a society where not everyone functions in the same way. Establishing causal links between behavioral deviations and specific genetic neurological deficits has proven difficult (35), and ADHD may therefore appear more as a social construction of deviant behavior (34).

Today’s discourse is characterized by a power structure in which the medical understanding of diagnoses is hegemonic and thus holds the authority to define reality (8–10, 18). The medical framework presents itself as an ostensibly objective lens through which behaviors can be categorized as desirable or undesirable, faultless or blameworthy. Yet conceptualizations of health and illness, and the diagnostic boundaries between normality and deviation, remain—despite their scientific grounding—human constructions that are not immune to heuristic biases or social processes.

### Being caught between normality and diagnosis

Beyond existing at the intersection of body and mind, the ADHD diagnosis is positioned squarely in the crossfire between stigma and support (36). Both the diagnosis’s ontological status and its normative consequences interact and contribute to the individual’s ongoing identity negotiation. A diagnosis may reduce social rejection (11), but it also risks carrying negative assumptions about the diagnosed one (11, 37, 38)—aspects the individual must navigate in their self-identity development. The sharp rise in ADHD diagnoses in Sweden over the past decade (21) has further fueled the debate about the diagnosis’s place in society. On the one hand, diagnostic prevalence is defended on the grounds that a diagnosis can provide essential support in daily life (39); on the other hand, it is criticized as an excessive medicalization of social difficulties (18, 31, 40).

Narratives about diagnoses have become increasingly accessible, giving laypeople a different position in discussions about diagnostic categories and in perceptions of what a diagnosis is (41). Several studies also indicate that the growing prevalence of health-related misinformation about ADHD may have triggered a trend of cyberchondria (42, 43), shaping identities not only among adolescents with an ADHD diagnosis but also among those with ADHD-like difficulties without formal diagnosis that self-diagnose based on readily available information (44). The ADHD phenomenon is therefore not solely a concern for the diagnosed, but also for those without an official diagnosis.

Captivity between “normality” and diagnosis refers to the lived experience of not living up the normate (33) nor the diagnostic criteria of ADHD. These adolescents can be understood as situated in a diagnostic borderland—at risk of extensive stigmatization on the basis of their ADHD-like behaviors while simultaneously being excluded from the support that accompanies a formal ADHD diagnosis. In addition to the complications arising from a categorical understanding of ADHD—particularly when young people fall just short of receiving a long-hoped-for diagnosis—this dynamic also represents a delicate balance between stigma and support that adolescents must traverse in their negotiations within the self.

### Conceptualizing the self

5 conceptualized the self as comprising two components. One component consisted of the original, spontaneous, and unique dimension of the human being, designated the *I*. The other component referred to the learned cultural and social dimensions of a person and was termed the *Me*. Mead argued that the *Me* represented the part of the self that was developed and formed within the social context. The *Me* enabled the self to comprehend, interact with, and define situations in light of the cultural symbols—particularly language, morals, values, meanings, and practices—that constituted a *Me* emerging from a specific social environment (5).

Concurrently, there was another dimension of the self, the *I*, which was active, acting, and situated in the present. The *I* was the ephemeral and acting agent of the self, while the *Me* encompassed the social roles or cultural characters that society reproduced and generated through the generalized other, which, broadly speaking, represented the social dimension of the self. Societal values were incorporated into the self through the *Me*, and each group to which a person belonged produced a particular *Me*, while other ideas were more broadly shared across society (5).

Humans therefore had both the generalized other—that is, society’s more general values—to take into account, as well as several institution- and group-specific *Mes* that coexisted, competed, conflicted, and influenced the emerging self. Through the values of society and groups, the *Me* exercised social regulation and control over the *I* (5).

This occurred through a role-playing process in which children and adolescents, as well as adults, learned roles or *Mes* through the play–game sequence. *Play* illustrated a situation in which the individual had not yet fully mastered the role and could move in and out of it, whereas *game* illustrated a situation in which the individual had fully learned to inhabit and perform a given role (5).

Thus, the emerging self is a lifelong process shaped by the dynamic interplay between the *I* and the *Me* and the implications of this interaction.

### The dialogical self

The emerging self may be most productively conceptualized as the dialogical self (7) in which subjectivity develops through dynamic dialogical processes between the *I* and multiple *I-positions* that populate the self and may be understood as different *Mes*. While this conceptualization is rooted in 5 foundational distinction between *I* and *Me*, it is through Hermans’ (45, 46) Dialogical Self Theory that the relationship between the *I* and its potential positions is explicitly theorized as fundamentally dialogical rather than oppositional.

Within this framework, the relation between the *I* and its various *Mes* may be marked by conflict, tension, cooperation, or alliance; however, these relational modalities are understood as surface manifestations of an underlying dialogical process (45, 46). Apparent fixations or binary oppositions within the self should therefore not be interpreted as static or mutually exclusive positions, but rather as outcomes of ongoing dialogical dynamics in which both *I* and *Me* are continuously shaped through mutual influence. The degree to which these dynamics are dialogical may vary, yet their dialogical nature remains constitutive of the self (46).

This dialogical understanding is here extended to the concept of the emerging self, and more specifically to the relationship between normality and ADHD as theorized in this article. From this perspective, subjectivity is not organized around a stable dichotomy between normality and deviation, but emerges through dialogical negotiations among multiple positions within the self. Such a view foregrounds dialogicality as a central organizing principle of meaning-making, through which individuals continuously relate to themselves, to others, and to culturally available discourses (7).

## Method

This study is part of a larger research project on ADHD, self, and identity among adolescents with an ADHD diagnosis and those without a diagnosis but who display ADHD-like behaviors. The project was approved by the Swedish Ethical Review Authority (reference number 2023-06162-01).

We employed a qualitative research interview method (47) to explore how the emerging self—conceptualized through 5—was shaped by the experiences of ten Swedish adolescents positioned between normality and diagnosis. In-depth interviews are particularly suitable for exploring participants’ experiences, feelings, and self-descriptions (47, 48).

### Participants

Participants were recruited primarily through convenience sampling. The study was advertised to guardians via social media (Facebook and Instagram) as well as through snowball sampling (49), in which participants could refer others who might be interested. Recruitment occurred nationally across Sweden. Potential participants registered their interest via an electronic link, which also provided written participant information outlining the purpose and structure of the study for both participants and guardians. Those who wished to proceed completed an electronic survey containing background questions (age, gender, country of birth, occupation, parental education level), questions about previous diagnoses (ADHD, autism, intellectual disability, psychosis, other), as well as rating scales for symptoms of ADHD, autism, depression, and substance use. The background questions were designed to describe the sample, while the diagnostic and symptom-related items served for inclusion and exclusion purposes.

The sample consisted of ten adolescents aged 15–18 years (six girls, three boys, and one individual who identified as non-binary). The participants embraced having ADHD-like difficulties and scored high on ADHD self-report scales but reported no formal ADHD diagnosis. Five participants self-reported symptom levels (i.e., rated “often” or “very often” on six or more symptoms within one or more domains) in line with a combined ADHD presentation, three in line with an inattentive presentation, one in line with a hyperactive/impulsive presentation, and one reported sub-threshold symptom levels (i.e., embraced fewer symptoms than the diagnostic threshold of at least six out of nine symptoms per domain). No clinical evaluation was conducted, and functional impairment was not assessed. Thus, the sample self-identifies with ADHD and it is not certain that they would qualify for a formal diagnosis. The sample scored low on autistic symptoms, depression, and substance use. The rating scales used for selection were established and psychometrically sound instruments: The Adult ADHD Self- Report Scale – adolescent version (50), the Montgomery Åsberg Depression Rating Scale (51), the Alcohol Use Disorders Identification Test (52), the Drug Use Disorders Identification Test (53), and the Autism-Spectrum Quotient (54).

Individuals meeting the inclusion criteria were contacted by telephone and provided with additional verbal information along with the opportunity to ask questions before scheduling an interview. To ensure informed consent, participants received a link to a digital consent form, which they completed prior to the interview. Those who did not meet the criteria, or who were otherwise not eligible, received a brief neutral e-mail stating that participation would not be possible, along with the option to contact the principal investigator for more information.

Ten interviews were deemed sufficient to achieve preliminary thematic saturation, allowing for the identification of recurring experiences and a diversity of perspectives within the scope of an initial exploratory investigation of this under-studied group. All participants were enrolled in upper secondary school or high school, resulting in an age range of 15–18 years. In a larger, non-exploratory study aimed at analytic generalization, a greater number of participants would reasonably be required to achieve a more stable and varied saturation.

All participants were assigned pseudonyms, and the results section begins with a brief summary of the participants at the group level. In **Appendix 2**, all participants are described individually.

### Interview methodology

The interviews were conducted by Author 2 (LY) under the supervision of Author 3 (MF). The semi-structured interview guide primarily consisted of open-ended questions, allowing for subjectivity, interpretation, and flexibility. It focused on experiences related to home, school, and leisure, while also allowing for follow-up questions. The interview guide is provided in **Appendix 1**, and the questions served as a common structure across all interviews.

To enable a geographically diverse sample, interviews were conducted digitally. All interviews were recorded to facilitate analysis. Each interview began with information about the purpose and structure of the study; participants were informed of their right not to answer specific questions, their right to withdraw at any time without explanation, and their right to revoke consent. Because many participants had no prior experience with research, particular care was taken to equip them with explicit language and strategies for expressing discomfort, requesting a break, or declining to answer.

Interviews were initially planned to last 60–90 minutes, with an average duration of 75 minutes. The total dataset amounts to 13 hours of interview material. When an interview was expected to exceed 60 minutes, participants were offered a break; they were informed of this beforehand and were given linguistic cues to request a break themselves. All interviews were transcribed verbatim.

### An abductive analytical coding

To facilitate data management and coding, interview transcripts were imported into MAXQDA, a widely used software package for qualitative and mixed-methods analysis. Data were analyzed using an abductive approach, and codes were developed and organized within the software.

An abductive approach involves moving between empirical observations and theoretical concepts to progressively develop the most plausible explanation of the phenomenon. Unlike deductive approaches, which begin from theory, and inductive approaches, which begin from data, abduction integrates both. The researcher begins with preliminary observations, tests them against relevant theoretical perspectives, and then returns to the empirical material to deepen or adjust the emerging interpretation. In this way, patterns and explanations emerge through a dialogue between data and theory (55–57).

An abductive strategy was used during the coding process to apply concepts from the self—specifically *I* and *Me* (5)—in order to conceptualize a shared understanding of what was meant by the self at the outset of the analysis. *I* and *Me* were used to understand how the self was shaped when positioned between normality and diagnosis.

Through abductive coding, the emerging self was gradually delineated: *I* was understood and coded as participants’ original and personal voices, thoughts, feelings, and responses to *Me*, which was constituted by influences from the surrounding social world. *Me*, in turn, manifested various social roles, narratives, and perspectives communicated by others—such as family members or institutional actors (e.g., school staff, organizational representatives)—that sought to discipline, guide, manage, or constrain *I*.

In the analysis of the interviews, the following dialogical themes were identified and coded as salient dimensions of the dialogue between the *I* and the *Me* within the emerging self:

- An *I* in dialogue with a medicalized *Me.*
- Resistance to stereotypical understandings of the diagnosis.
- An ADHD diagnosis as a pathway toward a positive reframing of the *I.*
- ADHD-like difficulties as a form of double stigma.

These dialogical themes are examined in greater depth in the Results section.

The coding was conducted by Author 2 (LY) in collaboration with Author 1 (JG).

## Results

The group of participants was characterized by a recurring experience of not fully fitting into the environments that surrounded them—school, leisure activities, family, and friendships. Despite substantial individual differences, they shared an underlying sense of being “different,” often tied to energy levels, impulsivity, social insecurity, or difficulties with concentration and demanding settings. Several described how they adjusted their behavior to meet the expectations of others: some toned down their energy to avoid taking up too much space, while others withdrew entirely to avoid the risk of being judged.

Many moved between two versions of themselves. One was more spontaneous, confident, and energetic in environments where they felt safe (which we conceptualized and coded as *I*). The other was more restrained, controlled, or cautious in school and other performance-oriented contexts (which we conceptualized and coded as different *Me*s).

The school environment emerged as particularly challenging for nearly all participants. Increasing academic demands, difficulties maintaining focus, getting stuck on tasks, and recurring feelings of inadequacy appeared in almost every narrative. In contrast, sports, creative pursuits, and hobbies were often described as spaces of freedom in which they could feel competent, strong, or comfortable. Parents or relatives with diagnoses, along with comments from teachers or school staff, frequently triggered thoughts about ADHD—either as a potential explanation, a source of support, or a possible obstacle. Throughout their accounts, the participants expressed a strong desire to better understand themselves and to be freed from the persistent feeling of being wrong, misplaced, or strange (for a concise case study description of each participant, see **Appendix 2**).

Being a young person with suspected ADHD meant navigating a delicate balance between individual agency on the one side and medical or institutional constraint on the other. Narratives related to ADHD could be both constructive and destructive for the adolescents’ emerging selves, and *I* responded to these tensions. In the results section, four central themes were presented to show how *I* and *Me* shaped one another within the participants’ emerging selves in relation to ADHD-like challenges.

### An *I* in dialogue with a medicalized *Me*

The interviewed adolescents demonstrated in various ways how they, through a medical understanding of the self, became medicalized. When this medical understanding was activated in relation to the adolescents’ *I*, it involved two aspects: the different contexts within which this understanding was communicated, and how *I* responded to it. The medical framing entered the adolescents’ lives through various interactional situations with others that activated different *Me*s, each tied to roles with differing possibilities for expressing how well the young people could meet role expectations. These interactions, combined with *I*’s response, meant that the adolescents adopted a medical understanding of their selves. Through this process of external–internal dialogue, an external medical framing came to shape their internal understanding of the emerging self as part of an ongoing dialogical process.

Veronica explained:

When I was little, I always felt different. I didn’t function the same way as everyone else. But it wasn’t really a deep thought I had until maybe a year ago [ … ] because during my teens I started noticing it even more clearly, like, “Wow, I don’t understand how these people can function like this,” and “I don’t get it … How can everyone walk around with a ten-step skincare routine when I can’t even manage to brush my teeth at night?” [ … ] And then I thought, “Okay, maybe there’s something going on with me.” So I started googling like crazy. Symptoms of autism or ADHD [ … ] But I don’t think I have ADHD. I mean, wouldn’t they have noticed earlier? But I read up on it, and a lot of it actually fits. And I’ve also had friends who’ve been like, “Yeah, you probably have some kind of diagnosis.” One of my friends asked me at school—she was like, “Do you have a diagnosis?” when I was talking nonstop during class, and I was like, “Ouch.” No, I’m kidding, I wasn’t offended. I actually thought it was funny because by then I had already started thinking about it. So I was like, “Oh, so you think so too.” And that kind of made me feel … I don’t know … like I was being confirmed in some way.

Ilona described how her parents and the school had brought forth a medicalized version of the *Me*.

I mean, my parents have pointed it out all the time—basically for as long as I can remember. Like, “Why can’t you focus on this thing?” or “Why can’t you focus on that?” and “Just sit down and study for half an hour now.” [ … ] Teachers have mentioned it too, at parent-teacher meetings and things like that … So it came a bit from school as well. And then my mom … because my cousin, the one who’s [age], he was just diagnosed with ADHD, and his mom thought that I should’ve gotten a diagnosis too, kind of. Yeah. So they were also pushing a bit.

These interactional situations were often combinations of exchanges with friends, relatives, professional expertise, and media. When these sources coexisted, they exerted a strong dialogical influence, shaping the adolescents’ understanding of their *I* and, ultimately, their emerging selves. A striking feature across many interactional situations described by the adolescents was that the medical understanding and the ADHD-role were invoked as a response to perceived shortcomings in their *I* when measured against role expectations. In many such contexts, when the medical framing was activated by others—professionals, friends, or family—it appeared in the form of care. The ADHD diagnosis was seen by the surrounding adults as a means of supporting the young person through difficulties, intended to help and guide.

Lisa, for instance, first encountered the medical framing when a special-education teacher in junior high administered screening questionnaires and then directly told her that she “had ADHD in some way.” Lisa had not perceived her difficulties as a deviation but had assumed that her functioning was normal and common. In her account, the ADHD-role preceded any experienced need; her teacher had offered Lisa a new *Me* through which to understand herself, prompting her to redefine herself and thus her self-concept. Institutional voices, alongside the medical framework and significant others from the social sphere of their lives, crystallized into a dominant dialogical coalition. The cumulative pressure of this coalition made it difficult for the I to withstand, thereby shaping the emerging self.

For several participants, friends and family repeatedly commented on how certain behaviors might be linked to an ADHD-role, even when the people making these comments had no medical authority.

August described how a parent had activated a medicalized understanding of his behavior:

It was fairly recently that I started thinking about it. Maybe a year ago or so. That’s when my [parent] was about to get a diagnosis for it. And then my [parent] started thinking like, “Well, you’re actually very much like me, so you might want to think about it too.” Yeah, it really fits very well with who I am as a person, the kinds of tendencies I have and so on.

Irma had long heard from friends and family about her suspected ADHD. They joked about her energy and, directly—though jokingly—insisted that she “really had ADHD.” Although such remarks were meant lightheartedly, they had a profound impact on Irma. She was forced, in moments of heavy self-reflection, to consider what increasingly felt like a fact: her understanding of her *I* strongly resembled the picture of ADHD she now held. This “no smoke without fire” reasoning appeared frequently in the transcripts and led many adolescents into an internal dialogue across their different positions.

In some cases, the adolescents themselves were more active agents in their medicalization. For Birk, the medical framing emerged through a sibling’s ADHD diagnosis. His own difficulties with focus and sporadic hyper-interests, in light of the sibling’s similarities, led him to believe that he too might “have ADHD”.

Birk explained:

I have a [sibling] with ADHD, and some of the things I see in them, I can recognize in myself as well … But it’s mainly the focus thing, like how you just start drifting off. And then when you’re interested in something, you get really interested. But also, when I was younger, it was hard for me to sit still. I was always running around, looking at things. But I think a lot of that is pretty similar for most young boys.

For Veronica, the internet became an important source of social information about ADHD and, in turn, about her own thoughts, feelings, and behaviors. Through online research, Veronica gained access to entirely new ways of understanding her *I*. Digital information further expanded what could be linked to *I*. In addition, Veronica’s friends reinforced claims to the ADHD-role through joking reminders that the label might be worth considering.

Although the medical understanding was communicated to Birk and Lisa primarily in the school environment, and to Veronica and Irma largely through friends, the central theme across these accounts of medicalized youth was normativity—the idea of the “normal adolescent.” Against this backdrop, a dialogical process emerged between *I* and *Me* centered on the question: “Do I have ADHD?”

### Resistance to a stereotypical understanding of the diagnosis

Several adolescents described forms of resistance to letting ADHD narratives rein in their *I* and define their emerging selves. This resistance stemmed from an experienced incompatibility between an externally imposed ADHD-*Me* and *I*. For many participants, the ADHD-role itself underpinned their resistance to an ADHD identity. Resistance emerged in response to an ADHD-*Me* that participants experienced as overly stereotypical and reductive, leaving little room for nuance or depth. While participants did report different types of symptoms, the issue was less one of binary categorization and more a perceived loss of complexity, in which ambition and ability could otherwise exist alongside aspects such as impulsivity.

The interview material revealed stereotypical images of “people with ADHD”—an impulsive troublemaker who throws things in the classroom, disrupts others, and creates chaos. While often acknowledged as outdated, this stereotype nonetheless influenced understandings of ADHD and thus impacted the emerging self in undesirable ways. Axel explained that teachers associated ADHD with “extroverted, outgoing, loud people,” which made him reluctant to adopt the label because he did not want them to attribute such traits to him. In school-based interactions, ADHD thus emerged not only as a role of disorderliness but also as a discrediting attribute one might wish to hide.

Veronica described a similar experience:

I’m still not 100% sure I have ADHD since I haven’t been diagnosed, but I’m pretty sure. I know it was pretty obvious in my early years. And my parents even joked about it … And if they had followed up on it and maybe not seen the stigma around it and been like, ‘no, not ADHD,’ but instead taken me for an evaluation and made me aware of it … Because I didn’t have that thought. It wasn’t even close when I was little. Back then I thought of a boy throwing chairs in the classroom. That was my image, and that wasn’t me.

The image of ADHD that Axel and especially Veronica relayed devalued and oversimplified people with ADHD. The stereotype lacked any mention of ambition, reflection, or multifaceted abilities. ADHD was reduced to largely indefensible behaviors—often impulsive aggression—that Veronica could not identify with and that Axel did not want to be associated with. The diagnosis was also gendered through these stereotypes: not only did Veronica not throw chairs—she was also a girl.

For many adolescents, joking references to ADHD indicated that the label carried a particular stigma. The primary framing was that ADHD was a discrediting attribute they did not possess; yet through jokes, a secondary framing emerged—suggesting they did possess this stigmatized trait. Many emphasized that these jokes were made “without meaning anything negative,” yet this repeated insistence on the diagnosis’s supposed harmlessness was contradicted by the very interactions that framed ADHD as a negatively charged deviation.

Birk described the jargon among friends in his free time:

It can easily get pretty messy, and you kind of … end up saying unpleasant things even if you don’t really mean them. That can happen at the same time as we still have a friendly tone, and we’re kind of honest with each other, and we don’t say anything that we know someone would actually get genuinely hurt by if we joked about it.

Irma described how laughter, as a social practice within her family, functioned as a way of defusing situations that deviated from what was expected:

For my family it has always been … if I’ve done something a bit strange, they’ve laughed about it together with me. They never laugh at me, they laugh with me, and that makes me feel like I can be myself.

Lovisa’s resistance, which involved rejecting ADHD as part of her self-concept, was rooted in the diagnosis’s stigmatizing implications. A formal ADHD diagnosis could also entail perceived limitations on future opportunities after compulsory schooling. Lovisa realized during a career-inspiration day in ninth grade that she was interested in military service and possibly working in the Armed Forces. Representatives told her she would fit well—”You can keep track of people and organize things.” At the same time, she believed the military was not interested in people with autism or ADHD, which would have shattered her plans had she been diagnosed earlier. Today she refused to adopt an ADHD diagnosis. She had long felt treated as a “problem child who needed fixing.” Being stigmatized over time, receiving formal confirmation that cemented such stigma, and facing reduced life opportunities as a result made the ADHD identity difficult for her to embrace.

### An ADHD diagnosis as a pathway to a positive reframing of the *I*

While ADHD was often associated with unrestrained impulsivity and disorder, a parallel and more positive image of the condition also existed. ADHD provided a formal recognition and an acceptable explanatory framework for the adolescents’ difficulties. As a form of symbolic equipment for the ADHD-role, the diagnosis improved their chances of being reframed from “problem children” to “fighters” in their emerging selves. This illustrated a power dynamic embedded in diagnostic practices, capable of reshaping how others perceive young people with ADHD-related difficulties. Diagnosis can reconfigure dialogical space in positive ways, supporting a more affirmatively charged development of the emerging self. Tensions arise, however, when individuals resist positioning their I in accordance with the medical framework’s authoritative claims, as such resistance risks being re-positioned as problematic behavior, for instance through labeling the individual as a “troublemaker.”

The ADHD-role’s ability to offer recognition was particularly evident in Veronica, who wanted to use ADHD to explain her academic performance:

Veronica explained:

When it came to oral tasks, I felt [ … ] that the focus should be on the language. So, if I had difficulty sticking to the topic, it had absolutely nothing to do with English; it had to do with how my brain functioned. So, then I thought my teacher ought to know that, so she could assess me fairly based on that. [ … ]. Otherwise, I think it was mostly because if I noticed that I was different from most people around me, then just getting a diagnosis and being able to say—if people asked “Okay, what are you doing?”—being able to explain it. Not just “No, I’m a strange individual.” No, being able to have something objective. “I have ADHD.” [ … ]. That’s just how I function.

Veronica described ADHD as something objective, using expressions such as “how my brain functions,” “mental,” and “different.” In doing so, she not only articulated a boundary between normality and deviation but also framed that boundary as biological. At the same time, the lines became blurry again when she suggested that having an ADHD diagnosis could distinguish her from being merely a “strange individual.”

Melissa used similar language when she described how her “brain shut down” when she was expected to do something she did not find interesting. In many adolescents’ accounts, agency was displaced from themselves to “the brain” rather than to their own controlled thinking. For them, ADHD became a new form of normalized *Me*, one that they expected others to meet with more tolerance and recognition than if they were simply perceived as “odd.”

Ilona described a shift in how she was perceived by a friend—from being seen as “stupid” because of her difficulties to being viewed as “normal” in the absence of any diagnosis—after she found the energy to engage more in her studies:

Now that things are going better for me in school, because I’ve been more interested, a friend from my ninth-grade class said, “I thought you were stupid, but it was just that you couldn’t really study back then,” after I explained it to her. It’s nice that someone knows I’m not stupid. I just didn’t have the energy to study, or whatever you’d call it.

Axel clarified how teachers might perceive ADHD-like difficulties, and how their perceptions can shift in the presence of a diagnosis:

I think they see it as something annoying, maybe. Especially if you don’t have it diagnosed, because then there’s no proof or anything. And then maybe they don’t believe you. They might just think you’re stupid. But if I had it and they saw my grades, then maybe they’d think, “Wow, he’s impressive—he has difficulties but still manages.” So it can go both ways.

Axel described several instances throughout the interview in which he had been repeatedly stigmatized by demanding teachers during his schooling. This stigmatization was based on a social framing of Axel, implying that his ADHD-like behaviors reflected a flaw in his personal character. Immediately, this exposed him to a high degree of personal blame and to the assumption that his difficulties were exaggerated or fabricated. Yet when he applied a medical framing instead, Axel suggested that the effect could be the opposite—that the ADHD-role in school might even impress teachers.

Axel spoke warmly about an ADHD diagnosis, believing it could have alleviated his difficulties, primarily because a diagnosis served as recognition from others that his struggles were real rather than made up. The teacher saw and acknowledged the student’s challenges, transforming the student’s weakness into something positive, something with developmental potential. Without a diagnosis, the student was seen as “stupid,” a “problem child,” or troublesome in other ways. The formal ADHD diagnosis thus served as essential symbolic equipment that allowed the adolescent to credibly inhabit the ADHD-role and to be recognized as a person with real difficulties.

### ADHD-like difficulties as a double stigma

Both medically and socially framed ADHD-like behaviors risked being stigmatizing, but if stigma was conceptualized as a discrediting relationship between role performance and role expectations, it followed that an ADHD *Me*—a role requiring a formal ADHD diagnosis—introduced new expectations for the adolescent that transformed previously unacceptable behaviors into understandable and tolerated ones. Active, energetic, and impulsive adolescents without a diagnosis were assessed in relation to other roles (for example, the student role), and thus a discrediting relationship between role performance and expectations emerged. This illustrates some of the challenges faced by individuals who experience symptoms but lack a formal diagnosis. Although they may resist simplified or stereotypical understandings of their capacities, they may still benefit from tailored pedagogical support. While diagnosis can facilitate both access to support and more positive perceptions, the provision of support without diagnosis may be perceived as unfair by peers.

For Lovisa, this meant dealing with classmates who felt she had unfairly received support given her lack of a formal ADHD diagnosis:

Lovisa recounted:

We were allowed to make some adjustments in school and postpone certain tests when I felt that I absolutely couldn’t take them. So they accepted it as it was. But at the same time, you would hear from classmates saying “It’s so unfair,” “You don’t have any paper saying you have ADHD,” “Why should you get it so easy?”

August described how he had previously struggled socially and how he hoped that receiving a diagnosis would serve as a kind of medical confirmation that his difficulties were due to clinical ADHD, allowing him to stop blaming himself:

I had a hard time with people, but I think it’s better now, kind of. I was bullied a bit in elementary and middle school, not in a brutal way, but I was the weird kid that no one wanted to hang out with. [ … ] Of course I hope I’ll be diagnosed, so I can be sure that this really is the problem and not just that I’m a bad person. I’m not particularly interested in being medicated, really. I just want to be sure that it’s the way I think it is.

In a similar vein, Kim portrayed herself as an incorrigible problem within the school:

I felt that since I … found things harder, or that it took me longer to read or to complete an assignment than everyone else, I started thinking that either the others were so, so much better than I was, or that there was something wrong with me. That it was … that *I* was the problem and that it wouldn’t get better no matter what I did … because it takes me longer to read a text than it does for my friend. Or it takes me longer to write a text than it does for my classmates.

Here, ADHD-like difficulties without a diagnosis continued to constitute a potential double stigma, as adolescents with such difficulties but without a formal diagnosis were recognized neither as good students nor as ADHD-persons. The adolescent therefore faced a choice—either to invest immense effort in schoolwork to claim the identity of a good student, with the looming risk of repeated stigmatizing failures, or to engage in a dialogue with the ADHD-self in order to be recognized as an ADHD-person. These relationships between normality and deviation formed the essence of what was referred to as the diagnostic borderland.

Participants in the study articulated the lived experience of being caught in a form of captivity between “normality” and diagnosis, a position that cannot be understood in binary either/or terms. Rather than simply being “normal” or “diagnosed,” they described themselves as inhabiting a dialogical in-between space in which their I was positioned neither fully within the normate nor within the diagnostic category of ADHD.

This diagnostic borderland was experienced as a liminal dialogical space marked by ongoing negotiations between multiple voices. Within this space, participants struggled with stereotyping, simplification, and reductive interpretations of who they were, alongside experiences of stigma and the risk of having their aspirations delegitimized. At the same time, some participants had encountered the productive potential of the diagnostic apparatus, whose authority could reconfigure dialogical relations in the social environment and generate recognition through new identity positions—such as positioning the self as a “fighter”—within the emerging self.

Importantly, access to support and exposure to stigma did not follow a clear either/or logic. Support could be both enabling and socially risky, particularly when provided in the absence of a formal diagnosis. Beyond the complications produced by categorical understandings of ADHD—especially when young people fall just short of receiving a long-hoped-for diagnosis—this situation illustrates a non-binary, tension-filled balance between stigma and support that adolescents must continually negotiate within the dialogical self.

## Discussion

At present, there is limited research on young people who display ADHD-related symptoms but, for various reasons, do not seek or receive a diagnosis. This article therefore contributes much-needed insight into this group. The reasons for the absence of a diagnosis may be several—for example, that they do not meet all diagnostic criteria, or that they themselves resist being diagnosed due to simplistic stereotypes surrounding ADHD and concerns about negative consequences for future career opportunities. A diagnosis can entail both desired and undesired effects (11, 37, 38, 58).

These young people shared many of the experiences documented in research on adolescents with a formal ADHD diagnosis. They reported challenges in social settings, in school, and in identity work shaped by gender and the self (12–20). Yet they differed in one crucial respect: they did not have a diagnosis.

Behaviors perceived as disruptive or problematic are typically understood through a medicalized conceptual apparatus, whereby individuals who do not conform to what is deemed normal, expected, and desirable are interpreted within a medical framework. As Bradby (8) notes, medical categories are never neutral; they are shaped by social processes, cultural assumptions, and institutional logics that determine what counts as deviance and which interventions are considered legitimate. Defining certain behaviors as medical problems simultaneously produces a set of expectations regarding how individuals should understand themselves—that is, their emerging self—and what forms of intervention institutions may consider necessary (e.g., healthcare, school, home). As a result, adolescents situated between diagnosis and non-diagnosis often find themselves in a complex zone of “dialogical tension” (7, 45, 46): they are interpreted through medical frameworks, yet do not gain access to the resources, explanatory models, or legitimacy that a formal diagnosis would provide. At the same time, the study shows that when pedagogical support is nonetheless offered, a kind of double stigma may arise, as peers may perceive such support as illegitimate and as conferring unfair advantages, for example in school.

Without a formal diagnosis, yet with experienced symptoms, participants occupied a diagnostic borderland or limbo, positioned between “normality” and diagnosis in ways that resist binary either/or interpretations (33). Rather than being simply normal or diagnosed, they inhabited a dialogical in-between space where the I was negotiated in relation to multiple, influential, and sometimes contradictory voices (45, 46). Within this space, participants struggled with stereotyping, simplification, and reductive interpretations of who they were, alongside experiences of stigma and the risk of having their aspirations delegitimized. At the same time, diagnosis could also function productively by reconfiguring dialogical relations in the social environment and enabling recognition through new identity positions within the emerging self. Importantly, access to support and exposure to stigma did not follow a clear binary logic: support could be both enabling and socially risky, particularly in the absence of a formal diagnosis. Taken together, this illustrates a tension-filled, non-binary balance between stigma and support that adolescents must continuously negotiate within the dialogical self.

Illich’s classic critique of the expanding power of medicine (1977) is particularly relevant in this context, not as an epistemological challenge to the scientific validity of medicine, but as a socio-political and institutional analysis of medicalization and professional authority. Illich argued that modern medicine increasingly colonizes everyday life through processes of social and cultural iatrogenesis, whereby professional systems come to define normative boundaries between health and pathology, competence and deviance. This expansion of medical authority does not merely produce diagnoses, but also reorganizes social expectations, moral judgments, and access to legitimacy and support.

From this perspective, adolescents without a formal diagnosis but with ADHD-like difficulties can be understood as occupying a particularly vulnerable position within medicalized social orders. On the one hand, they are subject to the same medicalizing discourses that shape how behaviors such as inattention, impulsivity, or restlessness are interpreted. On the other hand, because they fall outside the formal diagnostic categories that regulate access to institutional recognition and support, they risk becoming socially and institutionally invisible. In Illich’s (9) terms, this illustrates how medical power operates not only through inclusion within diagnostic categories, but also through exclusion from them.

Importantly, the absence of a diagnosis does not imply the absence of medical influence. Rather, these adolescents may experience a specific form of marginalization, in which their experiences are implicitly evaluated through medical norms without granting them the protections, accommodations, or legitimacy that formal diagnosis affords. In this sense, Illich’s (9) critique helps illuminate how medicalization functions as a structuring force in social life, shaping subject positions and opportunities even for those who remain formally undiagnosed.

A critical understanding of the institutional power of medicine makes it possible to see how this group of adolescents is situated within a system where deviance is rapidly translated into medical terms (8, 9, 11, 31, 34, 40). This process produces a medicalized *Me* that is expected to manage and discipline the original, spontaneous *I*, which is perceived as ill-fitting within the socially designed environments they inhabit (5). This creates tension and friction in the emerging self, as the original *I* risks being interpreted as negative, blameworthy, and stigmatized through the ways in which society responds (4). A diagnosis, however, can alter society’s perception of the previously blameworthy *I*, as the young person’s efforts—for example in school—and any resulting improvements may then be reinterpreted as signs of being a “fighter” or a “hero.” Without a diagnosis, this perceptual shift becomes difficult to initiate, as a person may, for various reasons, develop or mature and begin to act in ways that align more closely with the *Mes* expected by society, school, or family. This raises important questions about how society defines deviance from expected *Mes*, whose voices hold interpretive authority, and which experiences risk being overlooked as medical knowledge plays an increasingly central role in shaping our understanding of young people’s lives.

The medical apparatus operates within a social context in which it is frequently used to interpret behaviors that fall outside the bounds of what is considered desirable (8, 9, 18, 34). Medicine must therefore be understood in relation to the social and cultural processes at work in society. In Sweden, ADHD diagnoses have increased dramatically over the past decade (21, 22). This may be linked to changes in the arenas where young people spend much of their time, particularly school (14, 17, 18, 20).

The Swedish school system is characterized by large class sizes and few teachers expected to manage heavy workloads. This can lead to a narrowing of the *Me*-role of “student”; it simply becomes more difficult to discipline the *I* in accordance with the school’s expectations of what the *Me* should be (14). When someone deviates, it may therefore appear natural to medicalize the behavior—owing to constrained roles, limited resources, overburdened teachers, and the powerful societal position of medicine (14, 18, 31, 40).

In addition, a recent review published in Sweden by the Inspectorate of Health and Social Care (IVO) revealed that many private companies conducting ADHD assessments for paying clients exhibit significant shortcomings and violate regulatory standards (59). In the review, all ten audited providers were found to have deficiencies and failed to meet the requirements for safe and high-quality care in relation to ADHD diagnosis and treatment. Taken together, these findings point to the existence of a market in which private actors, in practice, sell ADHD diagnoses for financial gain, driven at least in part by commercial incentives rather than strictly medical considerations (8, 60, 61).

How this emerging market for ADHD diagnosis, and its entanglement with the organization of the Swedish school system, compares to other national contexts lies beyond the scope of the present study. Nevertheless, such dynamics must be considered integral to any critical social-scientific understanding of the role and positioning of ADHD diagnosis within the Swedish context.

Young people with ADHD-symptoms but without a formal diagnosis may be particularly vulnerable in such a situation. Smaller classes, where teachers are better able to support students, would likely benefit these adolescents and reduce tension and friction between *Me* and *I* in the emerging self. A reorganization toward smaller class sizes may be a viable path forward, as it would allow for increased pedagogical interaction and more time per pupil, thereby enhancing flexibility in the role expectations that define what is considered normal (14). From a societal perspective, however, there is a need for deeper reflection on parental expectations and anxieties around children’s performance, and for broadening these understandings. When the *Me*-role becomes too narrow—defined largely by performance and compliance—more children and adolescents are likely to be medicalized when they deviate. Such a shift could strengthen the dialogue and cooperation between *Me* and *I* in the emerging self, as the situation itself could foster a new pedagogical and social climate in classrooms, reflected in the society of mind (7).

Additionally, it is important to challenge the notion that only students with formal diagnoses require support. According to the Swedish Education Act (62), educational support should be provided based on students’ needs rather than the presence of a diagnosis. Consequently, there may be a need both for informational efforts to clarify the intentions of the legislation and for work aimed at establishing a non-stigmatizing understanding that students without diagnoses may also have special educational needs within the Swedish school context.

The findings of the present study indicate that students without diagnoses, but who nonetheless experience difficulties, do receive educational support. At the same time, the results suggest that prevailing assumptions among both teachers and peers contribute to this support being perceived as stigmatizing. In some cases, a formal diagnosis is sought in order to legitimize support measures, a practice that runs counter to the intentions of the Education Act (36).

Further research should examine the prevalence of the challenges reported by participants in this study, as well as deepen our understanding of the experiences of adolescents who exhibit ADHD-like difficulties but lack a formal diagnosis. A neurodiversity perspective (32) may aid individuals that feel different, yet for various reasons to not want to pursuit a diagnosis or do not fulfill all criteria, to integrate aspects of themselves that are well-functioning with aspects that are challenging and do not adhere to the narrow norms of being for instance, a good student.

This study does not claim to be exhaustive; rather, it represents an initial step toward a more nuanced understanding within the Swedish context.

## Data Availability

The original contributions presented in the study are included in the article/**Supplementary Material**. Further inquiries can be directed to the corresponding author.
