# Supplementary material for: Between “normality” and diagnosis: strains between the I and the Me in undiagnosed adolescents with ADHD symptoms
Source: Front Psychiatry. 2026 May 25;17:1771510. doi: 10.3389/fpsyt.2026.1771510 (PMC13243204; doi:10.3389/fpsyt.2026.1771510)
Supplement: Supplementary file 1 [file Table1.docx]

**Interview Guide**

**General questions exploring narrative identity from a present-day perspective**

1. Could you start by telling me a bit about yourself—who you are and what you enjoy doing in your free time?
2. Why do you think you are the way you are, and why do you think you enjoy these particular things?
3. If you reflect for a moment, do you notice any difference between the person you feel you are and the person you would like to be?

**General questions exploring narrative identity in relation to ADHD**

1. You do not have an ADHD diagnosis, but you experience symptoms or difficulties that you feel point to ADHD. Could you tell me a bit more about this (how you first noticed it, how old you were when you began to think this might be the case, whether others—parents, school, friends—have commented on it, whether you have undergone an assessment and why/why not, and how this has influenced how you see yourself and how others see you)?
2. Have you received any specific support at school or any treatment from Child and Adolescent Psychiatry (BUP) because of this? Would you like to tell me more about that?
3. Is there anything you think could have been done differently?

**More focused questions exploring identity constructions in relation to school**

1. How do you perceive your school experience? If it helps, you may divide it into different periods (e.g., primary school, lower secondary, upper secondary, university/college, etc.).
2. During your schooling, have you ever felt that you became someone other than the person you want to be or feel you are?
3. Would you like to tell me a bit more about that?
4. How have you managed this clash or discrepancy?
5. What do you think could have been done to avoid this?
6. In what ways has this affected your relationships with classmates and teachers?

**More focused questions exploring identity constructions in relation to leisure activities**

1. How do you feel your free time and any leisure activities have been? If it helps, you may divide it into different periods or activities you have participated in.
2. In your free time or during leisure activities, have you ever felt that you became someone other than the person you want to be or feel you are?
3. Would you like to tell me a bit more about that?
4. How have you managed this clash or discrepancy?
5. What do you think could have been done to avoid this?
6. Are there any contexts where you feel you are allowed to be exactly who you are or become the person you want to be?
7. Would you like to tell me a bit more about that?

**More focused questions exploring healthcare’s influence on identity construction**

1. If you have had contact with Child and Adolescent Psychiatry (BUP): How do you feel that contact with BUP/psychiatric services has worked for you?
2. Would you like to describe how your contact with BUP has affected you?
3. Is there anything you think could have been done differently?

**More focused questions exploring identity constructions in relation to other people**

1. How do you perceive your relationships with other people over time? If it helps, you may divide this into periods (e.g., early childhood, lower secondary, upper secondary, university/college, etc.).
2. Have you experienced that, in relation to others, you became someone other than the person you want to be or feel you are?
3. Would you like to tell me a bit more about that?
4. How have you managed this clash or discrepancy?
5. What do you think could have been done to avoid this?
6. Are there other contexts in which you have experienced similar things? Please feel free to describe them.
7. How have you managed this clash or discrepancy?
8. What do you think could have been done to avoid this?
9. Are there relationships where you feel you are allowed to be exactly who you are or become the person you want to be?
10. Would you like to tell me a bit more about that?

**Concluding questions**

1. Is there any question I should have asked to understand you better?
2. Is there any question you would like to return to before we finish?
