# Supplementary material for: Between “normality” and diagnosis: strains between the I and the Me in undiagnosed adolescents with ADHD symptoms
Source: Front Psychiatry. 2026 May 25;17:1771510. doi: 10.3389/fpsyt.2026.1771510 (PMC13243204; doi:10.3389/fpsyt.2026.1771510)
Supplement: Supplementary file 2 [file Table2.docx]

**Appendix 2: Short case descriptions of the participants**

**“Veronica”**

Throughout her childhood, Veronica has been described—by others as well as by herself—as energetic. In her family, she is seen as the extroverted, cheerful, and spontaneous daughter, but among her friends, a shift in personality occurs. For a long time, Veronica has felt different from her peers. *“I don’t function the same way as everyone else,”* she emphasizes, describing how this sense of being different led her, during middle and lower secondary school, to withdraw more and more from social interactions. Fearing judgment, she stopped answering questions openly in class, stopped asserting herself among her friends, and avoided seeking out new relationships. She became increasingly withdrawn, and she describes herself during this period as a particularly difficult person to handle. She blames herself for occasional conflicts with friends, as she believes she came across as passive-aggressive.
When Veronica’s mother noticed these personality changes during her teenage years, they decided to seek support from Child and Adolescent Psychiatry (BUP). When the psychologist at BUP suggested that Veronica displayed extensive symptoms of ADHD, many pieces finally fell into place for her.

**“Kim”**

Kim is the child of two parents who are leaders in an outdoor organization. Kim jokingly describes it as “bad luck” to have parents with such an interest, but this upbringing has nevertheless resulted in a close relationship both with the parents and with nature. The love of the outdoors—and of dogs—became a space where Kim felt free and could do whatever felt right in the moment. There, Kim could fully be the person they have always been: tough, strong, and energetic.
School, however, was difficult for Kim and therefore came second. In sixth grade, during the period when academic demands increased, Kim began meeting with the school counselor, who was the first to point out Kim’s difficulties. In lower secondary school, questions about Kim’s identity emerged—*Who was Kim?* Kim realized they were non-binary and also began to suspect they might have ADHD, which led Kim and their parents to seek an assessment through Child and Adolescent Psychiatry.^[[1]](#footnote-1)^

**“August”**

August feels bizarre, strange, and different. Throughout his early school years, he was labeled *“the weird kid,”* and he experienced being avoided by other students. He had a small group of friends and could be himself with them, but due to early bullying, he now thinks carefully before interacting with anyone outside this circle. He adjusts his behavior to avoid making others uncomfortable.
This persistent feeling of being *“strange”* has led him to believe something must be wrong with him. When one of his parents was diagnosed with ADHD a year ago, the idea emerged that he might also have ADHD. He believes that a diagnosis would give him an important explanation for his behavior and difficulties—one that might free him from the feeling of being *“a bad person.”*

**“Irma”**

Irma has always felt close to the people she knows best. She is creative like one parent and spontaneous like the other. Her friends match her energy perfectly, and with them she feels at home. She enjoys spending time doing different activities together. She does not feel she often becomes someone completely different in relationships, but she also acknowledges that one cannot behave however one wants around people who aren’t close. In those situations, she tones down her energetic side to fit in.
As a social person, Irma has found it difficult in school to stay away from her classmates when she is expected to focus on lessons. She wishes she could work in a more structured way. With many relatives diagnosed with ADHD, Irma and her family have reflected on her declining academic performance and increasing concentration difficulties, connecting them to a potential ADHD diagnosis.

**“Ilona”**

For a long time, Ilona has been reminded by her parents and teachers that she is not performing well enough in school. She personally finds school very difficult—especially in lower secondary school, when expectations increased and classes focused on subjects she found uninteresting. In upper secondary school, she enrolled in a vocational program, where she feels her strengths finally have space to shine.
Ilona adores her little cousins and younger sister—and the feeling is mutual. Children do not judge her in the same way her parents, teachers, BUP, or some teenagers do. Ilona thrives when she is allowed to be herself and does not have to worry about being judged or seen as a failure. Environments that place too much pressure on her, or make her feel inadequate, she tends to leave in favor of spaces free from friction.

**“Lovisa”**

Lovisa is cheerful, energetic, and fiercely competitive. During matches in her ball sport, she describes herself as *“a bit crazy”*: she shouts at her teammates, organizes the game, and acts as a cheerleader to motivate the team toward victory. She enjoys this role but acknowledges that things can easily go wrong, and she sometimes feels guilty for scolding teammates when they are not focused. Regardless of whether the match or practice goes well or poorly, her energy level is always high.
School, however, has been challenging in recent years. In the early grades, school posed no problem, but as expectations increased in lower secondary school, she struggled her way through. She freezes on assignments, has difficulty concentrating, and often cannot finish tasks on time. Her teachers have encouraged her to quit volleyball to focus on school.
Lovisa has considered whether she might want an ADHD diagnosis, believing it would help teachers and classmates understand her better. But she quickly concludes that it is too late, as she believes a diagnosis would ruin her dream of joining the military.

**“Lisa”**

Lisa is an active upper secondary student. In her free time, she has balanced the demanding environment of competitive basketball with the freedom of artistic activities. She has played piano, sung, and pursued various creative expressions. In all these contexts, she has enjoyed herself most when she can simply *be*—when she can just *do*. When her basketball coach set expectations too high, Lisa quit organized basketball and instead played informally with friends. She wanted the freedom to do things her own way.
Although Lisa sees herself as quick to learn, she has had difficulties in school. Through her special education teacher’s observations, she began to suspect she might have ADHD. Reading and writing had long been very difficult for her, and she always preferred creative and social forms of schoolwork, such as engaging group assignments. Early in upper secondary school, she was diagnosed with dyslexia, which not only entitled her to school accommodations but also gave her a new way of understanding herself.
Even though Lisa has sometimes been shy and withdrawn—due to school changes and conflicts with classmates—she still sees herself as a social person who occasionally hesitates to show her social side for fear of being perceived as annoying.

**“Melissa”**

Melissa is often torn between being the shy girl at school and the energetic, outgoing girl at the stables. As an enthusiast of an animal-related activity, she is open, lively, and cheerful—largely because it is an environment where help is readily available and expectations align more closely with the person she wants to be.
In school, however, she sees herself as shy because she fears doing or being something wrong. She avoids answering questions aloud and also avoids asking questions, fearful of being labeled *“stupid.”* What stands out about Melissa is that she views her reserved nature as a strength, as it helps her blend in more easily with her friends. She does not want to take up space—she just wants to be.

**“Axel”**

Axel’s parents have always been meticulous about their son’s upbringing. Axel performs well both academically and in sports, and he sees himself as someone who likes to do things properly. At the same time, he notices tendencies that make him think he might not be like everyone else. When he leaves the classroom to use the bathroom and get a much-needed break, teachers question him and clarify their expectations. When he tries to engage in class by asking or answering questions, he risks being labeled as stupid.
Axel describes himself as impulsive, active, and social, enjoying spending time with friends—especially through a particular sport. One parent resembles him, and after starting their own assessment, encouraged Axel to undergo one as well. His assessment did not progress very far, but he still wonders whether ADHD could be part of who he is.

**“Birk”**

Much like his parents, Birk dislikes sitting still and doing nothing. His sibling has ADD, and using them as a reference point, Birk has wondered whether he might also have a psychiatric diagnosis. In school, he has struggled to concentrate—especially when a teacher has failed to build a personal connection with him or engage his interests. He attributes his socially difficult years in middle school to the school environment.
He underwent an evaluation for dyslexia, resulting in a formal diagnosis, and the accommodations that followed have been helpful. At the same time, he still wonders whether there might be something more than dyslexia shaping his everyday life.

1. Kim was asked at the end of the interview which pronouns they preferred; therefore, the pronouns they/them are used throughout when referring to Kim. [↑](#footnote-ref-1)
